# Lifeguards: A Forgotten Aspect of Drowning Prevention

**DOI:** 10.5249/jivr.v2i1.32

**Published:** 2010-01

**Authors:** David C Schwebel, Heather N Jones, Erika Holder, Francesca Marciani

**Affiliations:** ^*a*^Department of Psychology, University of Alabama at Birmingham, USA.; ^*b*^Department of Psychology, Oberlin College, USA.

## Abstract

**Abstract:**

An alarming number of drownings occur in lifeguarded swimming areas, where one might presume swimmers are protected from injury. One reason drownings occur in lifeguarded swimming areas is because lifeguard surveillance is a highly difficult task. Observational research suggests lifeguards are usually alert, but researchers also report egregious examples of inattention. We offer three strategies that have initial empirical support to reduce risk of drowning at lifeguarded swimming areas: (a) regular training to help lifeguards recognize they are vulnerable to drowning events and to raise their confidence; (b) regular practice via simulated emergency responses, and (c) addressing staff schedules so lifeguards can devote full attention to protecting swimmer safety while on duty. ‎

## Introduction

Drowning is among the leading causes of death worldwide. Children are especially vulnerable. In the United States, over 1000 children ages 0-18 are killed each year through drowning.^1^ Many of those deaths are attributable to poor adult supervision. Infants are left in bathtubs unsupervised. Newly mobile toddlers wander off and drown in pails, toilets, or tubs. Preschoolers discover ungated swimming pools and drown. Other drownings, including many to older children and adults, occur at un-guarded public swimming areas, such as beaches, rivers, and residential swimming pools. A small but alarming number of drowning deaths, however, occur at public swimming areas that are guarded by professional lifeguards. According to one industry document, roughly a third of drowning deaths in the United States, over 500 individuals annually, occur at lifeguarded pools.^2^

**How can deaths in lifeguarded swimming areas be prevented?**

Deaths in lifeguarded swimming areas are preventable. Anecdotal evidence in most cases suggests the lifeguard did not notice the individual struggling or drowning. On the surface, it seems implausible that someone could die while swimming in a location where one or more individuals are hired to prevent such events. Consider, however, the task of a lifeguard. He or she must sit for long hours, often in hot weather, watching for a very rare event to occur. A large body of scientific literature documents the incredible cognitive and perceptual challenge this presents to human beings. Frequently conducted in the context of screening baggage for explosives at airports, there is strong evidence that humans simply are not very good at noticing rare events while completing a boring, repetitive task.^3-5^ With lifeguards, the situation is compounded by the fact that most are young, fairly inexperienced, and poorly paid. As adolescents and young adults, many lifeguards are developmentally unprepared to handle the responsibility of their positions.^6^

Observational research on lifeguard behavior has documented a tremendous amount of high-quality, professional work.^7-8^ In one report, for example, lifeguards were attending to their assigned area 91% of the time.^8^ This is encouraging. However, one should not overlook the corollary to the finding: It also suggests that lifeguards were not attending to potential dangers 9% of the time.

Just documenting the problem will not change it, of course. The aquatics industry is acutely aware of the challenges they face to prevent drownings in lifeguarded swimming areas. Behavioral scientists have much to offer the aquatics industry. Most prominently, behavioral scientists should address the question of how one can help lifeguards complete the highly challenging task of identifying rare events (drownings) while completing a repetitive scanning task in an often uncomfortably hot, sunny environment. It is a multifaceted problem that will require a multifaceted solution, but below we propose three initiatives that have initial empirical support, that are relatively easy to implement, and that may immediately contribute to a reduction in death at lifeguarded public swimming areas in the United States and worldwide.

First, lifeguards must attend regular meetings and training sessions. In one report, a single brief training session, held mid-summer, increased lifeguard surveillance and decreased swimmer risk-taking during a summer swimming season.^7^ Such sessions should be developed with scientific knowledge of health-related behavior change in mind. Lifeguards should feel vulnerable: They should recognize that drownings can and do occur. Training also should help lifeguards overcome barriers they perceive in completing their job successfully. Problem-solving and confidence-raising are important steps toward high-quality surveillance of swimming areas.

Second, lifeguards must practice emergency responses. A large body of literature confirms the efficacy of frequent role-playing to improve individuals’ performance under stressful emergency situations.^9-10^ Many aquatics centers have begun conducting “audits” whereby a dummy is floated on the water, or a shadow dropped to the bottom of the water, and lifeguards must immediately react as if a real emergency has occurred. One would expect such audits might increase lifeguards’ feelings of susceptibility to drowning emergencies, as well as increasing their confidence and honing their skills to deal with them.

Third, pragmatic organizational issues must be addressed. Most American swimming areas operate with limited budgets. Resources must be funneled to protect safety, at all other costs. Lifeguards should not be expected or permitted to clean swimming pools, accept admission money, or perform other duties while also monitoring the safety of swimmers. Regular rotations, frequent breaks, and adequate staffing are absolutely essential for lifeguards to properly complete their duties. Such strategies have been used to reduce fatigue effects on conveyor belts^11^ and are also implicated by research in fields such as driving safety^12^and fatigue in athletic competitions.^13^ Without implementation of these and other logistical initiatives, we cannot expect lifeguards to properly complete their work.

## Conclusions

In summary, observational and empirical research suggests lifeguard surveillance is generally high quality, but subject also to potentially fatal lapses. During unannounced research-related visits to public swimming pools, we have witnessed lifeguards talking on the phone, reading magazines, and cleaning the pool. We have seen lifeguards playing with child swimmers, talking to other lifeguards, and moving deck furniture. We have seen lifeguards working while obviously ill or fatigued. Together, behavioral scientists and public health specialists must partner with the aquatics industry to stop such behaviors and to identify and implement strategies that will reduce risk and prevent drownings at lifeguarded swimming areas.
